# The Benefit of Additional Oviposition Targets for a Polyphagous Butterfly

**DOI:** 10.1673/031.007.0301

**Published:** 2007-01-18

**Authors:** Josefin Johansson, Anders Bergström, Niklas Janz

**Affiliations:** Department of Zoology, Stockholm University, 106 91 Stockholm, Sweden

**Keywords:** specialist, generalist, host plant range, oviposition preference hierarchy, Polygonia c-album

## Abstract

While the reasons for the prevalence of specialists over generalists among herbivorous insects have been at the focus of much interest, less effort has been put into understanding the polyphagous exceptions. Recent studies have suggested that these exceptions may be important for insect diversification, which calls for a better understanding of the potential factors that can lead to an increased host plant repertoire. Females of the Nymphalid butterfly, *Polygonia c*-*album*, were used to test if egg output and/or likelihood of finding a host increased with the addition of a secondary host. There was no effect of prior eggs on the host for willingness to oviposit on a plant. The main experiments were conducted both in small laboratory cages and in large outdoor experimental arenas. No positive effect was found when another oviposition target was added in small cages in the laboratory. On the other hand, in the outdoor arenas the females more often found a host to oviposit on and had a higher egg output when they had access to an additional host, even though the second host was lower in their preference hierarchy. The difference between these experiments was attributed to searching for acceptable host plants within a patch, a factor that was included in the large cages but not in the small. When host availability is limited, adding oviposition targets can potentially act to counterbalance specialization and thus favor the evolution of generalization.

## Introduction

The predominance of relative resource specialization among plant-feeding insects has prompted an intense interest in the processes and circumstances that can act to limit the range of plants used as hosts. A number of possible factors have been suggested, including trade-offs in feeding efficiency (Joshi and Thompson 1995; Traxler and Joern 1999; Agrawal 2000), predation (Bernays and Graham 1988), and neural constraints on oviposition behavior (Bernays and Wcislo 1994; Bernays 2001).

Even though host specialization is a general rule among plant-feeding insects (Futuyma and Moreno 1988; Thompson 1994), the rule is not without exceptions, even among otherwise highly specialized groups of insects such as the butterflies (Nylin and Janz 1999; Janz et al. 2001). These exceptions can be evolutionarily important as there are now indications that host expansions may have played an important role in the impressive diversification of plant-feeding insects (Janz et al. 2001; 2006; Weingartner et al. 2006). It would seem then, that there is a need to understand not only the rule, but also its exceptions. Unfortunately, our understanding of the mechanisms behind host expansion is poor. Bernays and Minkenberg (1997) suggested two major reasons for being a generalist: diet mixing and greater resource availability. While the possibility of obtaining a more balanced mix of nutrients may be important for insects with browsing life-styles, many insects are parasitic on their hosts, meaning that, even if they have the capacity to feed on multiple hosts, each individual larva will only experience one host species (and often individual) during its life (c.f. Price 1980; Thompson 1982). For such parasitic insects, diet mixing is not ecologically realistic, and also appears to have little positive influence on larval growth and survival (Bernays and Minkenberg 1997). This prompted Bernays and Minkenberg to suggest that, for these insects, the more universal advantage of greater resource availability and versatility should be more important.

This shifts the perspective from the larva towards the female, as the advantage of increased resource availability is mainly capitalized on during the search for oviposition sites. There is now good support for the view of oviposition behavior as a prime determiner of host plant range (e.g. Siemens et al. 1991; Resetarits 1996; Janz and Nylin 1997; Thompson 1998; Kuussaari et al. 2000; Nylin et al. 2000; Janz 2005). However, the effect of incorporating additional hosts is not necessarily positive. First, if the additional host is not of equal value, ovipositing on it would typically result in lower offspring fitness. Moreover, as each new plant requires a new set of abilities for searching and evaluation, multiple host use results in reduced accuracy, or increased decision time during oviposition (Janz and Nylin 1997; Bernays 1998; Bernays and Funk 1999; Nylin et al. 2000; Janz 2003b). Hence, if the net effect of adding additional targets for oviposition is to be positive, the increased encounter rates need to balance out not only the potentially lower larval performance on these additional plants, but also the effects of reduced accuracy and decision time. While it is clear that plant availability does affect host and patch preference on larger spatial scales (Thomas and Singer 1987; Kuussaari et al. 2000; Hanski and Singer 2001; Janz et al. 2005), direct evidence for the advantage of incorporating a host into the repertoire is still lacking. Considering the important role that host expansions may play in the diversification of plant-feeding insects (Janz et al. 2006), this lack of knowledge is troublesome.

This study investigates this most basic of possible advantages of a host range expansion: the benefit of increased availability of oviposition targets (Bernays and Minkenberg 1997). The problem with host expansion is that the added host may not be of equal value, in which case the additional oviposition targets would result in lower offspring fitness. The fundamental hypothesis is that adding oviposition targets, even when they are of lower quality, will be beneficial in terms of increased egg output and realized fecundity. However, testing this is not as straightforward as it may seem. A specialist could presumably compensate for the smaller number of oviposition targets by an increased efficiency in finding their single host plant, e.g. because they have been able to develop more accurate searching behaviors. Consequently, it can be hard to demonstrate such a benefit by comparing a generalist with a specialist species. The question must be asked, and tested, by looking at a given species with its current search abilities, and ask what effect the inclusion of additional host species of lower quality has. (This excludes true specialists, as they would not recognize any other plants as oviposition targets). The advantage of adding additional targets for oviposition should also be most pronounced in species that are time limited rather than egg limited, Time limitation will arise when either the favored host is rare, the female has a short predicted life span, or in environments with a short window of time for oviposition, In such situations, females will have difficulties laying their full load of eggs and there will be a selective advantage of adding additional targets for oviposition, as it would increase the chances of finding sufficient amounts of acceptable larval hosts during a female's life, This also calls for an experimental arena that can present a more challenging search situation for the ovipositing female, something that an oviposition trial in small lab cages does not do as the actual search phase is to a large extent excluded.

To this end, females of the polyphagous butterfly, *Polygonia c-album* L. (Nymphalidae), were used, They are capable of using a range of hosts in addition to the preferred plants in Urticales, but where the willingness to do so varies greatly between populations (Nylin 1988; Janz and Nylin 1997; Janz 1998; Nylin et al. 2000; Nylin et al. 2005). Both simultaneous choice trials in small lab cages and experiments in large outdoor arenas were used to be able to test the effect of scale and search complexity. The experiments were designed to mimic the natural situation when an additional host species contributes to increased numbers of oviposition targets, but where these will be of lower value. The two main predictions were, first, that adding an alternative host of lower quality (and by this, increasing the number of oviposition targets) results in an increased egg output in a situation when host plants are difficult to find. Second, that females confined to a small cage, where finding hosts is not an issue, should not show a corresponding increase in egg output when an additional oviposition target of lower quality is provided. Finally, a basic assumption of the experimental design was also tested: that an ovipositing female will not adjust her preference when conspecific eggs are already present on the plant.

## Materials and Methods

### Study species

This study was conducted on the palearctic butterfly *Polygonia c-album*, a member of the tribe Nymphalini (Nymphalidae). Its main habitat is open woodland and wood edges. Even if this adult-overwintering butterfly is robust and capable of surviving long periods in the adult stage, the available time for oviposition in spring can be short, especially in the northern parts of its distribution, where spring weather can be cold and unpredictable. Females normally lay their eggs singly or in small “clutches” of two to five eggs (Bergström et al. 2006), but have a capacity to lay several hundreds of eggs during their life-time. They are therefore more likely to be time limited than egg limited. The favored hosts are *Urtica dioica* (stinging nettle), *Humulus lupulus* (common hops), and *Ulmus glabra* (Wych elm) (Nylin 1988; Janz et al. 1994). Further down in the preference hierarchy are, for example, *Salix* spp. (willow), *Ribes* spp. (current), and *Betula pubescens* (downy birch). This preference hierarchy correlates well with offspring performance on the population level (Janz et al. 1994). The larvae can initiate and complete development on all of these hosts and the different populations seem to have similar preference hierarchies, but differ greatly in specificity (Nylin 1988; Janz 1998; Janz 2003a; Nylin et al. 2005), Some populations of *P. C*-*album* use only *U*. *dioica* as hosts and others, such as the one used in the present study, use all plants in the hierarchy. Fitness consequences of the hosts used in this study are relatively well known, through a series of studies aimed to specifically disentangle the preference-performance relationship. While adult weight can be higher on *Salix*, all other fitness components are higher on *Urtica*; *Urtica*-reared individuals have a higher growth rate, a shorter development time, a higher survival, more protein in the spermatophores, and are more prone to direct development (Janz et al. 1994; Nylin et al. 1996; Wedell *et al.* 1997). We have found that larval growth rate is the performance component that best summarizes offspring fitness in this system, and shows the best correlation with female preference (Janz et al. 1994; Nylin et al. 1996).

### Larval rearings

Experimental individuals originated from eggs oviposited by butterflies that were wild-caught in May 2003–2005 in the Stockholm area. The larvae were reared two by two in small plastic containers on *U*. *dioica* in environmental chambers. First they were reared in 17°C and a 12/12 (L:D) light regime, and after molting to the third larval instar they were transferred to an environment with 26°C and a 22/2 (L:D) light regime, a process known to induce direct development (Nylin 1989), After eclosion, all butterflies were placed in mating cages, approximately 1 × 1 × 1 m. They were provided with nectar plants and sponges with a solution of sucrose and water, and were monitored for mating.

**Figure 1.**
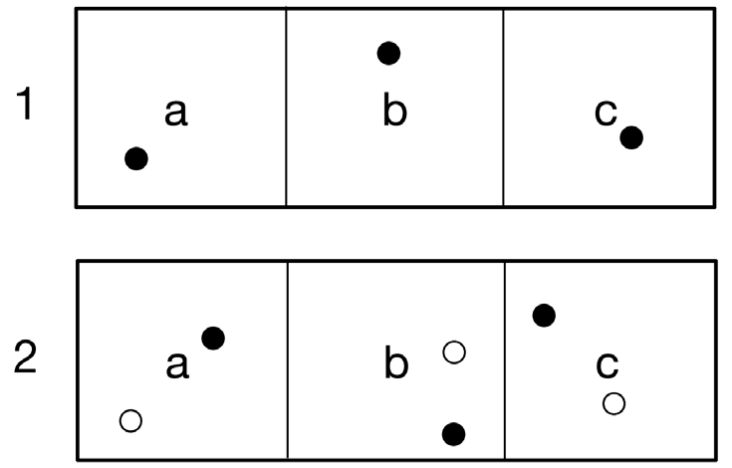
Schematic illustration of the outdoor flight cages. Two cages were divided into equally sized compartments (W × L × H: 8 × 10 × 4 m). Each trial was paired, so that a female receiving the one-host treatment in cage 1a was paired with a female receiving the two-host treatment in cage 2a. The treatments were shifted between cages after each trial. Filled circles represent a stalk of the primary host (*U. dioica*) and unfilled circles represent a stalk of the secondary host (*S*. *caprea*).

### Small cage experiments

These trials were conducted on second-generation butterflies during the years 2003 and 2005. Naive mated females were transferred to smaller oviposition cages (0.5 × 0.5 × 0.5 m) in a laboratory of Stockholm University, where the host plant choice experiment was conducted. The cages (wooden frame with sides and rear covered with cloth) were illuminated between 9.00 and 15.30 by 75W light bulbs, hanging approximately 40 cm above the transparent cage roof. The light bulbs also provided an additional source of heat. The floor was covered with moist paper and the females were supplied with diluted sucrose.

### Egg assessment preference trial

This experiment was performed to assure that the females' willingness to oviposit on a plant was not affected by prior eggs on the plant (Blaakmeer et al. 1994; Anbutsu and Togashi 1996). This would interfere with experimental design in that it would cause the female to spread her eggs between the plants more than she would in a natural situation. 21 mated, naive females were presented individually with a simultaneous choice of two stalks of stinging nettle (*U. dioica*), standing in water. The stalks were similar in all respects except for the presence of 20 conspecific eggs on one of the stalks. Females were allowed to oviposit during one day, between 9.00 and 15.30, and plant stalks were exchanged for new ones every second hour during this time to keep the different treatments as constant as possible across the length of the experiment. The difference in total number of eggs was analyzed with the Wilcoxon matched-pair signed-rank test.

### Oviposition targets preference trial

This experiment was designed to investigate if the females would benefit from having more than one host species in the cage, so that they were able to lay more eggs. 26 females were flown individually in these cages for the oviposition trials. Two different treatments were used, each lasting for two days. One treatment with only one stalk of *U*. *dioica* (one-host treatment), and the other treatment with two stalks, one each of stinging nettle and goat willow (*Salix caprea*) (two-host treatment). The stalks were cut to the same size and kept in bottles of water and they were exchanged upon signs of senescence. All females were given both treatments, and every second female started with the two-host treatment and the rest started with the one-host treatment. Eggs were removed from the plants and counted after each day. The plants were presented to the butterflies at equal distance from the central light and food sources. Data were analyzed with a paired t-test, and were log-transformed to satisfy the assumptions of the test.

### Large cage experiment

This experiment was carried out at the Tovetorp research station, 100 km south west of Stockholm during July 2003 and 2004. There were two large cages of oblong shape with half-circle shaped cross-sections. The cages had an east-westerly orientation and were located in an open pasture. They were covered with fine-meshed net that reduce solar radiation by approximately 25%. The bottom of the cages consisted of natural grassland vegetation, cut to about 0.2–0.3 m. Each cage was divided into three equally sized compartments by walls of insect net, each compartment was W × L × H: 8 × 10 × 4 m in size. The host plants were placed in bottles of water and there was also a commercial butterfly feeder (“Flutterby” from Nature Products) with artificial nectar in each compartment. The compartments were also equipped with twigs of non-host plants, to create a more challenging environment, and stems of additional nectar plants placed in bottles.

**Figure 2.**
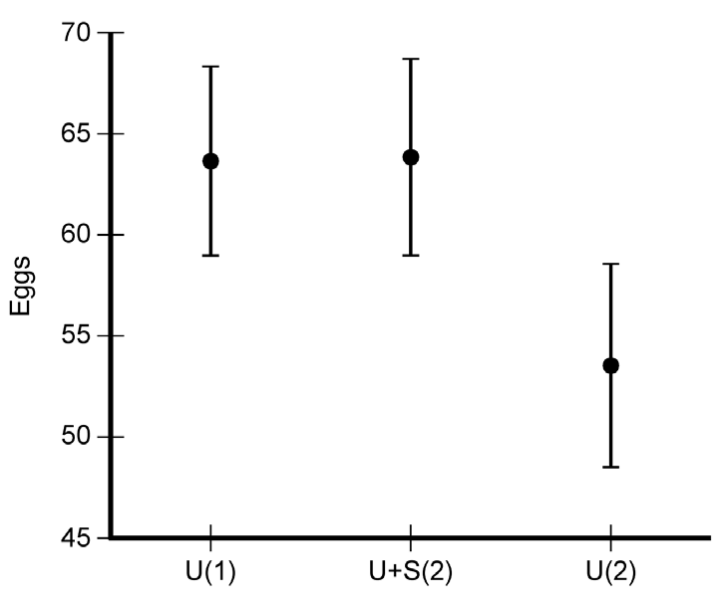
Number of eggs oviposited in the small cage trial by individual females of *P*. *c-album.* U = *Urtica dioica* (primary host), S = *Salix caprea* (secondary host). Treatments are marked by 1U = one host (*U*. *dioica*), U+S(2) = two hosts (*U. dioica* + *S. caprea*), U(2) = *#* of eggs laid on *U*. *dioica* in the two-host compartment. Females laid an equal number of eggs totally in both treatments and, as a consequence, the number of eggs laid on the primary host decreased when a secondary host was present. Means ± SE.

Each female was given one of two treatments; either one display each of *U. dioica* and *S. caprea*, cut to the same size, or with just one display of *U*. *dioica*. The plants were positioned randomly in the cage, with at least one meter to the cage net and, in the two-host treatment, at least two meters between the plants. Females were released in pairs, one female in each pair in each treatment, to control for effects of weather. The pairs consisted of the corresponding compartments in the two cages (compartment a, b or c; Fig. 1). Trials lasted for one day (10.00 AM to 17.00 PM) and treatments were shifted after each trial to control for cage effects. As we had no prior knowledge of how well the female could remember the position of a found host plant the data were analyzed in two ways. First, assuming that her spatial memory allows her to return to a host plant once it is found, we analyzed the data with the sign test, without taking the number of eggs laid into account. Hence, here we only measured whether a female found a plant to oviposit on or not during the time of the trial. Second, assuming that she cannot remember the position of a found host plant, we analyzed the data with the Wilcoxon matched-pair signed-rank test. Here, the total number of eggs laid in the two treatments was used as a measure of how often a plant was found to oviposit on. If none of the females in a comparison laid any eggs, the data were excluded from the analysis.

## Results

### Small cage experiments

#### Egg assessment preference trial

There was no effect of previous conspecific eggs on a plant. The females laid an equal number of eggs on the stalks with eggs as on the stalks without eggs (Wilcoxon signed-rank matched-pairs test, N = 21, z = -0.174, p = 0.862). Hence, the assumption in the following trials, that a plant will not be less preferred when eggs are present, appeared to be justified.

### Oviposition targets preference trial

As predicted, the addition of a lower quality host species did not result in an increased egg deposition in the small cages (Figure 2). They laid an average of 63.7 eggs/day in the one-host treatment and 63.8 eggs/day in the two-host treatment (paired t-test, N = 26, t = -0.0024, p = 0.998).

**Figure 3.**
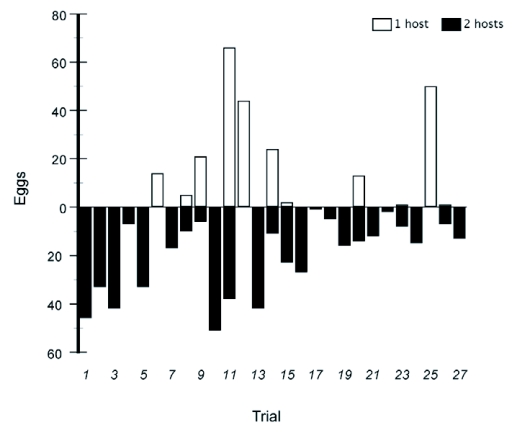
Total number of eggs laid by individual females of *P. c*-*album* in the two treatments in a paired design. Unfilled bars denote one-host treatment: one stalk of primary host only (*U*. *dioica*) and filled bars denote two-host treatment: one stalk of primary host (*U. dioica*) and one stalk of secondary host (*S*. *caprea*). Each trial consisted of two females who were flown simultaneously in one of two adjacent arenas, differing only in treatment.

Since the total number off eggs did not differ between treatments, and as females oviposited on both hosts when available, they must have spread their eggs across the hosts in the two-host treatment, even though the better host was always present and available. Indeed, when they had access to both *U. dioica* and *S. caprea*, the females appeared to lay fewer eggs on *U*. *dioica* than when they only had *U*. *dioica* in the cage (Figure 2). While a female laid an average of 63.7 eggs/day in the one-host treatment (where only *U*. *dioica* was available), they laid 53.5 eggs/days on *U*. *dioica* in the two-host treatment (paired t-test, N = 26, t = 1.9804, p = 0.059).

### Large cage experiment

In contrast to the small cage trial, the females in the large experimental arena did have a higher total egg output, and they also found a host more often, when they had more than one host plant species in the cage (Figure 3). Assuming that a female's spatial memory does not allow her to relocate a previously found host, the total number of eggs laid during the trial should be a good measure of search success. Under this assumption, there was a significantly higher number of eggs laid in the two-host treatment (Wilcoxon matched-pairs signed ranks test, N = 27, z = 2.019, p = 0.044). In 21 of 27 comparisons the females laid more eggs then when they had two instead of one host plant in the cage. If instead it is assumed that females will be able to relocate a previously found host by remembering its position, the number of times where the female was able to find a host and oviposit at all during the trial can be measured. Under this assumption, the difference between treatments was even stronger. In 24 of 27 cases the females in the two-host treatment found and laid eggs on a host, compared with the one-host treatment where 11 out of 27 females found the host and laid eggs (Sign test, N = 27, p = 0.006).

Total egg output was higher in the small cages, an average of 63.8 eggs/day, than in the large cages, where they laid an average of 13.3 eggs/day (Two-sample Wilcoxon rank-sum (Mann-Whitney) test; N = 26,54; z = 6.73; p<0.0001).

## Discussion

A basic assumption of the experiments in this study was that a female's willingness to accept a plant for oviposition should not be affected by conspecific eggs already on the plant. When this assumption was tested, no difference was found in willingness to oviposit on plants with and without eggs. This was not surprising, as females of *P*. *c*-*album* typically lay their eggs singly, occur in relatively low densities, and use hosts that are either large (most hosts are trees or shrubs) or often grow in large stands (such as *U*. *dioica*). Hence, in the field a female is not likely to encounter many plants with eggs already present and there should not be any selection for avoiding eggs during oviposition.

The main experiments were divided into a laboratory trial in small oviposition cages and an outdoor trial in large experimental arenas that required the female to search for host plants under more challenging conditions. Following predictions, females in the small cage trial did not show an increased egg output in the two-host treatment compared with the one-host treatment (Fig. 2). As the search phase was reduced in these trials, the deposition of eggs was mainly a result of decisions to land on a plant and to accept it for oviposition following landing. Consequently, they were probably able to reach their physiological limit of egg output in both small-cage treatments. If the total number of eggs laid remained constant in these treatments, and the females used both hosts for oviposition, a logical consequence is that they must have laid fewer eggs on the favored host when a less favored host was also present in the cage. This is actually a bit puzzling, as it would seem to make more sense to always use the best available host for oviposition. As eggs already on the plant do not affect oviposition decisions, why are females consistently laying eggs on lower-quality plants when a better plant is present? Clearly, the situation in these cages does not adequately reflect the natural situation of an ovipositing female, and the most fundamental aspect that is missing is that she does not have to search for plants. If finding plants is difficult, it would make sense to accept lower-ranked plants to some degree, upon encounter. We have previously suggested that accepting a plant for oviposition is probably not an all-or-none response, but rather that plants have associated probabilities of acceptance, that is dependent on preference rank (Janz et al. 2005). Hence, an adaptive response to encountering a lower-quality host would be to accept it for oviposition, albeit with a lower probability of acceptance, which would lead to a pattern of egg deposition similar to what was found in the small-cage experiment.

In the more complex outdoor experimental arena, where females did have to search for plants to oviposit on, the result was different. In this experiment, total egg output was significantly lower than in the small cages, indicating that they did not reach their physiological limit of daily egg output. Here, adding a lower-ranked plant to the arena resulted in an increased egg deposition, and a higher likelihood of succeeding in finding a plant to oviposit on during the time of the trial (Figure 3). Hence, there was a positive effect on oviposition whether it was assumed that a female can or cannot remember the position of a found host plant in the experimental arena. If finding a preferred host is difficult, adding an extra host species means more targets for oviposition and consequently an increased likelihood of finding acceptable hosts. It appears that this could potentially favor the inclusion of an additional host species into the repertoire. However, the net effect will depend on a range of ecological, as well as life-history factors, such as search efficiency, time available for oviposition, relative abundance of the plants involved and their different effects on larval performance. Inclusion of an additional host will be favorable when the most acceptable host is rare, when finding a host is difficult, and when the increased output of eggs outweighs the loss in fitness caused by laying eggs on a sub-optimal host.

As mentioned in the Introduction, it has turned out to be difficult to understand the mechanisms behind host expansions. We provide here a demonstration of one of the most basic potential advantages: the higher resource availability that comes with adding another host to the repertoire (Bernays and Minkenberg 1997). The puzzle of host expansions largely remains, however, and it is clear that much work will be needed to fully understand the mechanisms behind them. Again, we would like to stress the importance of understanding these processes better, as they have been suggested to play a key role in the diversification of plant-feeding insects (Janz et al. 2001; 2006; Weingartner et al. 2006).

## References

[bibr01] Agrawal AA 2000 Host-range evolution: Adaptation and trade-offs in fitness of mites on alternative hosts. *Ecology* 81 500 508

[bibr02] Anbutsu H Togashi K 1996 Deterred oviposition of *Monochamus alternates* (Coleoptera: Cerambycidae) on *Pinus densiflora* bolts from oviposition scars containing eggs or larvae. *Applied Entomology and Zoology* 31 481 488

[bibr03] Bergström A Janz N Nylin S 2006 Putting more eggs in the best basket: clutch size regulation in the comma butterfly. *Ecological Entomology* 31 255 260

[bibr04] Bernays EA 1998 The value of being a resource specialist: behavioral support for a neural hypothesis. *American Naturalist* 151 451 464 10.1086/286132 18811319

[bibr05] Bernays EA Graham M 1988 On the evolution of host specificity in phytophagous arthropods. *Ecology* 69 886 892

[bibr06] Bernays EA Wcislo WT 1994 Sensory capabilities, information processing, and resource specialization. *Quarterly Review of Biology* 69 187 204

[bibr07] Bernays EA 2001 Neural limitations in phytophagous insects: implications for diet breadth and evolution of host affiliation. *Annual Review of Entomology* 46 703 727 10.1146/annurev.ento.46.1.703 11112184

[bibr08] Bernays EA Minkenberg OPJM 1997 Insect herbivores: Different reasons for being a generalist. *Ecology* 78 1157 1169

[bibr09] Bernays EA Funk DJ 1999 Specialists make faster decisions than generalists: experiments with aphids. *Proceedings of the Royal Society of London Series B*-*Biological Sciences* 266 151 156

[bibr10] Blaakmeer A Hagenbeek D Vanbeek TA Degroot AE Schoonhoven LM Vanloon JJA 1994 Plant-response to eggs vs host marking pheromone as factors inhibiting oviposition by *Pieris brassicae*. *Journal of Chemical Ecology* 20 1657 1665 10.1007/BF02059887 24242658

[bibr11] Futuyma DJ Moreno G 1988 The evolution of ecological specialization. *Annual Review of Ecology and Systematics* 19 207 233

[bibr12] Hanski I Singer MC 2001 Extinction-colonization dynamics and host-plant choice in butterfly metapopulations. *American Naturalist* 158 341 353 10.1086/321985 18707331

[bibr13] Janz N 1998 Sex-linked inheritance of host-plant specialization in a polyphagous butterfly. *Proceedings of the Royal Society of London*, *Series B*-*Biological Sciences* 265 1675 1678

[bibr14] Janz N 2003a Sex-linkage of host plant use in butterflies. Boggs C.L. Ehrlich P.R. Watt W.B. *Butterflies*: *Ecology and Evolution Taking Flight* 229 239 Chicago University of Chicago Press

[bibr15] Janz N 2003b The cost of polyphagy: oviposition decision time vs error rate in a butterfly. *Oikos* 100 493 496

[bibr16] Janz N 2005 The relationship between habitat selection and preference for adult and larval food resources in the polyphagous butterfly *Vanessa cardui* (Lepidoptera: Nymphalidae). *Journal of Insect Behavior* 18 767 780

[bibr17] Janz N Bergströom A Johansson J 2005 Frequency dependence of host plant choice within and between patches: a large cage experiment. *Evolutionary Ecology* 19 289 302

[bibr18] Janz N Nylin S 1997 The role of female search behaviour in determining host plant range in plant feeding insects: a test of the information processing hypothesis. *Proceedings of the Royal Society of London Series Biological Sciences* 264 701 707

[bibr19] Janz N Nylin S Nyblom K 2001 Evolutionary dynamics of host plant specialization: a case study of the tribe Nymphalini. *Evolution* 55 783 796 11392396 10.1554/0014-3820(2001)055[0783:edohps]2.0.co;2

[bibr20] Janz N Nylin S Wahlberg N 2006 Diversity begets diversity: host expansions and the diversification of plant-feeding insects. *BMC Evolutionary Biology* 6 4 16420707 10.1186/1471-2148-6-4 PMC1382262

[bibr21] Janz N Nylin S Wedell N 1994 Host plant utilization in the comma butterfly: sources of variation and evolutionary implications. *Oecologia* 99 132 140 10.1007/BF00317093 28313958

[bibr22] Joshi A Thompson JN 1995 Trade-offs and the evolution of host specialization. *Evolutionary Ecology* 9 82 92

[bibr23] Kuussaari M Singer M Hanski I 2000 Local specialization and landscape-level influence on host use in an herbivorous insect. *Ecology* 81 2177 2187

[bibr24] Nylin S 1988 Host plant specialization and seasonality in a polyphagous butterfly, *Polygonia c*-*album* (Nymphalidae). *Oikos* 53 381 386

[bibr25] Nylin S 1989 Effects of changing photoperiods in the life cycle regulation of the comma butterfly, *Polygonia c*-*album* (Nymphalidae). *Ecological Entomology* 14 209 218

[bibr26] Nylin S Janz N 1999 Ecology and evolution of host plant range: butterflies as a model group. Olff H Brown V.K. Drent R.H. *Herbivores*: *Between Plants and Predators* 31 54 Oxford Blackwell

[bibr27] Nylin S Nygren GH Windig JJ Janz N Bergström A 2005 Genetics of host-plant preference in the comma butterfly Polygonia c-album (Nymphalidae), and evolutionary implications. *Biological Journal of the Linnean Society* 84 455 765

[bibr28] Nylin S Bergström A Janz N 2000 Butterfly host plant choice in the face of possible confusion. *Journal of Insect Behavior* 13 469 482

[bibr29] Nylin S Janz N Wedell N 1996 Oviposition plant preference and offspring performance in the comma butterfly: correlations and conflicts. *Entomologia Experimentalis et Applicata* 80 141 144

[bibr30] Price P.W 1980 *Evolutionary biology of parasites* Princeton Princeton University Press

[bibr31] Resetarits WJ 1996 Oviposition site choice and life history evolution. *American Zoologist* 36 205 215

[bibr32] Siemens DH Johnson CD Woodman RL 1991 Determinants of host range in bruchid beetles. *Ecology* 72 1560 1566

[bibr33] Thomas CD Singer MC 1987 Variation in host preference affects movement patterns within a butterfly population. *Ecology* 68 1262 1267

[bibr34] Thompson J.N 1982 *Interaction and coevolution* New York Wiley

[bibr35] Thompson J.N 1994 *The coevolutionary process* Chicago University of Chicago Press

[bibr36] Thompson JN 1998 The evolution of diet breadth: Monophagy and polyphagy in swallowtail butterflies. *Journal of Evolutionary Biology* 11 563 578

[bibr37] Traxler MA Joern A 1999 Performance tradeoffs for two hosts within and between populations of the oligophagous grasshopper Hesperotettix viridis (Acrididae). *Oikos* 87 239 250

[bibr38] Wedell N Nylin S Janz N 1997 Effects of larval host plant and sex on the propensity to enter diapause in the comma butterfly. *Oikos* 78 569 575

[bibr39] Weingartner E Wahlberg N Nylin S 2006 Dynamics of host plant use and species diversity in *Polygonia* butterflies (Nymphalidae). *Journal of Evolutionary Biology* 19 483 491 16599924 10.1111/j.1420-9101.2005.01009.x

